# Effect of osteoarthritis and its surgical treatment on patients’ quality of life: a longitudinal study

**DOI:** 10.1186/s12891-023-06662-w

**Published:** 2023-06-30

**Authors:** Gyöngyi Anna Mezey, Edit Paulik, Zsuzsanna Máté

**Affiliations:** Department of Public Health, Albert Szent-Györgyi Medical School, University of Szeged, 10 Dóm Square, Szeged, Hungary 6720

**Keywords:** Osteoarthritis, Quality of life outcomes, Total hip replacement, Total knee replacement, WHOQOL-BREF, WOMAC

## Abstract

**Background:**

Osteoarthritis (OA) is one of the primary causes of pain and disability worldwide leading to patients having some of the worst health-related quality of life (QOL). The purpose of our study was to investigate the progression of the generic and disease-specific QOL of osteoarthritic patients going through total hip or knee replacement surgery and the factors that might alter the effect of surgery on QOL.

**Methods:**

A longitudinal study was performed based on data collected from 120 OA patients who filled in the short version of the WHO’s generic measure of quality of life (WHOQOL-BREF) and the disease-specific Western Ontario and McMaster Universities Osteoarthritis Index (WOMAC) before and after surgery.

**Results:**

Domains related to physical health status showed relatively lower scores in patients before surgery. Patients reported a significant increase of QOL after surgery in the WHOQOL-BREF physical domain, especially if they were from the younger group (< 65 years, p = 0.022) or had a manual job (p = 0.008). Disease-specific QOL outcome results indicate that overall patients gained significantly better QOL in all domains of the WOMAC score. Patients with hip OA seemed to have the most benefit of their operation as they reported better outcome in WOMAC pain (p = 0.019), stiffness (p = 0.010), physical function domains (p = 0.011) and total score (p = 0.007) compared to knee OA patients.

**Conclusion:**

There was a statistically significant improvement in all domains concerning physical functions in the study population. Patients also reported significant improvement in the social relationship domain, which indicates that OA itself as well as its management might have a profound effect on patients’ life beyond the reduction of their pain.

## Background

Osteoarthritis (OA) is one of the primary causes of pain and disability worldwide. From 1990 to 2019, the disabilty-adjusted life year of hip osteoarthritis increased from 0.46 million to 1.04 million, reflecting a total increase of 126.97% [1]. The number of OA cases, increasing with age and obesity rates and showing female predominance reached 527.81 million cases globally in 2019; therefore, it remains a major public health concern [2, 3].

The pain and disability caused by OA are associated with articular cartilage degeneration and functional restrictions [4]. Resulting from the latter, OA also affects social connectedness, relationships, and emotional well-being, thus reducing multiple aspects of quality of life (QOL) [5]. Furthermore, knee OA has been shown to be significantly associated with deteriorated mental health [6].

Health is consistently regarded as an important aspect of QOL. Health-related QOL (HRQOL) aims to measure QOL components impacted by certain diseases and effectiveness of treatment. Therefore, studies on HRQOL may evaluate the quality and outcome of health care provided or may identify applicative items [7]. Analysis of QOL data can also identify subgroups, can help guide interventions to improve the situation of those with poor perceived health and avert more serious consequences [8]. Patients with chronic musculoskeletal pain have some of the worst HRQOL with severe restrictions in their work and daily living [9]. The WHO has developed a generic measure of QOL (WHOQOL) that encompasses general, physical, psychological, social, and environmental aspects, making it ideal for measuring a broad range of factors, thus giving a more complete picture of the individual’s life and wellbeing [10]. Reis et al. have used the abbreviated version of the WHOQOL assessment tool (WHOQOL-BREF) when reporting on how significant knee pain in elderly women with knee OA affected their balance and overall QOL compared to elderly women with no OA [11]. This decline in QOL has been supported by the study of Cavalcante et al. as well [12], and even younger patients (< 50 years) have reported a poorer QOL because of OA [13].

Hip and knee joint replacement surgery is regarded as one of the most successful operations in medicine as a whole [14], leading to statistically significant improvement in QOL by 4% after 6 weeks and 13% after 6 months [15]. Post-surgical improvements in pain and function have been shown to extend over years, but examining the whole spectrum of QOL might give a more in-depth understanding of outcomes relevant for the individual [16].

While measuring of generic QOL is advantageous when assessing the overall burden of a given health problem, disease-specific measures of QOL have the advantage of being frequently more responsive and clinically useful than generic measures by measuring the frequency and severity of specific symptoms [17]. Since its initial validation [18], the Western Ontario and McMaster Universities Osteoarthritis Index (WOMAC) has become a popular patient reported outcome measure used for evaluation of hip and knee OA. It has been used extensively in research studies [19–21] and clinical trials [22–24] and has been recognized by the Outcome Measures in Rheumatoid Arthritis Clinical Trials group (OMERACT) and Osteoarthritis Research Society International (OARSI) [25, 26]. It also can also be used to classify patient satisfaction after total knee arthroplasty [27].

The aim of our study was to investigate the progression of the generic and disease-specific QOL of osteoarthritic patients undergoing total hip or knee replacement surgery, and the factors that might alter the effects of surgery effect on QOL.

## Methods

### Study design and participants.

**Fig. 1:**
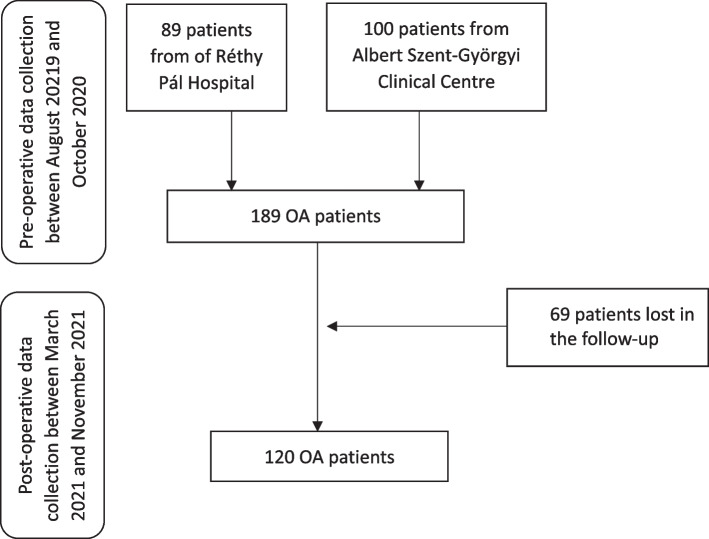
Flow diagram describing recruitment and progress of participants to the follow-up study

This longitudinal study was performed based on data collected from OA patients at the Department of Orthopaedics, Albert Szent-Györgyi Clinical Centre, University of Szeged (Szeged, Hungary) and at the Orthopaedic Ward of Réthy Pál Hospital of Békés County Central Hospital (Békéscsaba, Hungary) between August 2019 and October 2020. The recruitment process is illustrated in Fig. 1. Patients with knee or hip OA scheduled for total joint replacement surgery were involved, while patients receiving unicondylar knee arthroplasty were excluded. No other exclusion criteria were set. Participation was offered to all eligible patients consecutively to reduce selection bias. The self-administered questionnaires were filled in by the patients 24 h prior to surgery. To assess the effect of the surgery effect on QOL, post-operative data collection was carried out one year after the surgery when the questionnaires were sent and returned by post because of the COVID lockdown.

### Questionnaire

The questionnaire comprised sociodemographic data (e.g., age and gender) and QOL measuring tools.

Based on their age, participants were divided into two groups split at the age of 65 as that is the age of retirement in Hungary, making this grouping suitable to assess the effect of the disease and the treatment on both the economically active and inactive population. The level of education was considered ‘low’ if the participants had no high school degree, ‘middle’ if they had high school degree, and ‘high’ if they had a college or university degree. Based on job profile, participants whose job was physically demanding (e.g., manual labour) formed the “manual” group, while those with intellectual work (e.g., desk jobs) formed the “non-manual” group. After calculating the body mass index (BMI) (weight in kilograms divided by height squared in meters), the participants were grouped based on the WHO recommendation, as underweight (BMI below 18.5 kg/m^2^), normal (BMI 18.5 kg/m^2^ to 24.9 kg/m^2^), overweight (25.0 kg/m^2^ to 29.9 kg/m^2^), and obese (over 30.0 kg/m^2^) [28].

General QOL was measured by the validated Hungarian version of WHOQOL-BREF [29], which measures QOL with 26 questions in 4 domains: 1, Physical health (activities of daily living, dependence on medicinal substances and medical aids, energy and fatigue, mobility, pain and discomfort, sleep and rest, and work capacity); 2, Psychological health (bodily image and appearance, negative feelings, positive feelings, self-esteem, spirituality/religion/personal beliefs, thinking, learning, memory, and concentration); 3, Social relationships (personal relationships, social support, and sexual activity); 4, Environment (financial resources, freedom, physical safety and security, health and social care: accessibility and quality, home environment, opportunities for acquiring new information and skills, participation in and opportunities for recreation/leisure activities, physical environment, and transport). There are also two separate questions that are asked specifically about 1, the individual's overall perception of their own health and 2, the individual's overall perception of their QOL. The answers were measured by a 5-point Likert scale. In accordance with the instructions of the WHOQOL-BREF manual [30], domain scores were calculated and then converted to a 0–100 scale, whereas the results of the two separate questions were left untransformed. The higher score represented better QOL.

In order to assess the disease-specific QOL, we used the validated Hungarian version of the WOMAC [31] Index Version 3.1 numeric rating scale (NRS), which covers 3 dimensions through 24 items: Pain (5 items) during walking, going up/down the stairs, lying in bed, sitting, and standing upright; Stiffness (2 items) after waking up and later in the day; and Function (17 items) going up/down the stairs, rising from sitting, standing, bending, walking, getting in/out of a car, shopping, putting on/taking off socks, rising from bed, lying in bed, getting in/out of the bathtub, sitting, getting on/off toilet, performing heavy domestic duties or light domestic duties. All items were assessed by using a 1–10 NRS, (1 = no pain/stiffness/difficulty to 10 = extreme pain/stiffness/difficulty) totalling 24–240, where higher scores indicated increased pain and decrease function [32]. The results of the individual domains as well as the total score were later standardised to a 0–100 scale.

### Statistical analysis

Data analysis was carried out using IBM SPSS (Statistical Package for the Social Sciences) version 27 (SPSS Inc., Chicago, IL, USA). Descriptive statistics including frequency, percentage, mean and standard deviation (SD) were performed to characterize the study sample. As the outcome measures had non-normal distribution, Wilcoxon tests were carried out to assess the difference of the QOL outcome measures pre- and post-surgery. To explore the role of independent variables in the results, subgroups were made based on gender, age, affected joint, BMI categories, work profile, level of education and the presence of a comorbidity. Subgroup analyses were carried out using mixed-design two-way repeated measures ANOVA. Statistical significance was set at p < 0.05.

### Ethical consideration

The study was approved by the Regional and Institutional Review Board of Human Investigations in University of Szeged, Hungary (ID: 4059) and conducted in accordance with the Declaration of Helsinki. All subjects provided written informed consent before the questionnaire.

## Results

### Participants

The characteristics of the patients who we reached during the follow-up (*n* = 120) are shown in Table 1. The mean age was 68.68 years and the majority (85.8%) of the participants was overweight or obese. More than two-thirds of the patients were women (69.2%).

**Table 1:** Characteristics of patients

*Characteristics*	*n (%)*
Gender	
Men	37 (30.8)
Women	83 (69.2)
Age groups	
< 65 years	31 (25.8)
≥ 65 years	89 (74.2)
Level of education	
Lower	44 (36.7)
Middle	45 (37.5)
Higher	31 (25.8)
Job profile	
Manual	54 (45.0)
Non-manual	66 (55.0)
Affected joint	
Hip	56 (46.7)
Knee	64 (53.3)
Comorbidity	
Reported	30 (25.0)
Not reported	90 (75.0)
BMI categories (kg/m^2^)	
18.5–24.9	17 (14.2)
25.0–29.9	36 (30.0)
≥ 30.0	67 (55.8)

### Quality of life outcomes

#### General QOL (WHOQOL-BREF)

The opening questions of WHOQOL-BREF demonstrated patients reporting a significant increase of the perceived QOL, where score increased from 3.30 ± 0.84 to 3.58 ± 0.69 (*p* = 0.002) and of satisfaction with their health, the score of which rose from 3.03 ± 0.84 to 3.31 ± 0.70 (*p* = 0.001) after surgery. QOL outcome results indicated that the patients had significantly better QOL compared to their previous state in the physical health and social relationship domain. On the other hand, the improvement in the psychological domain was negligible, which can be attributed to the fact that the baseline values of that domain were better than those of the other domains (Table 2).

**Table 2:** Population results of the general QOL assessment

WHOQOL-BREF			
	Preoperative data	Postoperative data	
	mean ± SD	mean ± SD	
Physical health domain	46.51 ± 15.53	61.04 ± 16.70	p < 0.001
Psychological domain	63.99 ± 14.97	64.40 ± 15.14	p = 0.762
Social relationships domain	54.59 ± 21.87	59.58 ± 16.79	p = 0.012
Environment domain	64.93 ± 15.52	65.88 ± 13.85	p = 0.494

While all patients reported a significant increase of QOL in the WHOQOL-BREF physical domain, sub-group analysis showed that younger patients (< 65 years) reported significantly better outcomes compared to older ones (*p* = 0.022). Patients in the manual job group reported significantly greater increase in the physical (*p* = 0.008), and psychological (*p* = 0.003) domains compared to the non-manual group. Regardless of the patients’ gender, age, level of education, job profile, BMI, affected joint or the presence of other diseases, a significantly better QOL scores were achieved in the physical health (*p* < 0.001) and social relationships domains (from *p* = 0.033 to *p* < 0.001) after the surgery (Table 3) which is in accordance with the data in Table 2.

**Table 3:** Sub-group analysis results of the general QOL assessment

		WHOQOL-BREF											
		Physical health			Psychological			Social relationships			Environment		
		Preop	Postop	Sig	Preop	Postop	Sig	Preop	Postop	Sig	Preop	Postop	Sig
		mean ± SD	mean ± SD		mean ± SD	mean ± SD		mean ± SD	mean ± SD		mean ± SD	mean ± SD	
Gender				**p**_**c**_** < 0.001**			p_c_ = 0.77			**p**_**c**_** = 0.033**			p_c_ = 0.459
	Male	45.92 ± 15.04	64.49 ± 17.02	p_i_ = 0.121	66.68 ± 14.51	67.11 ± 15.57	p_i_ = 0.99	56.46 ± 21.84	59.95 ± 16.34	p_i_ = 0.610	65.46 ± 14.65	67.00 ± 14.55	p_i_ = 0.772
	Female	46.77 ± 15.82	59.51 ± 16.42		62.80 ± 15.10	63.19 ± 14.88		53.76 ± 21.96	59.41 ± 17.09		64.70 ± 15.98	65.37 ± 13.58	
Age groups				**p**_**c**_** < 0.001**			p_c_ = 0.548			p_c_ = 0.102			p_c_ = 0.477
	< 65	44.87 ± 16.62	66.13 ± 15.91	**p**_**i**_** = 0.022**	65.97 ± 16.21	67.97 ± 15.42	p_i_ = 0.487	62.94 ± 22.47	63.87 ± 16.43	p_i_ = 0.222	67.45 ± 14.68	68.84 ± 11.83	**p**_**i**_** = 0.036**
	≥ 65	47.08 ± 15.19	59.27 ± 16.68		63.30 ± 14.55	63.16 ± 14.92		51.69 ± 21.01	58.08 ± 2.59		64.06 ± 15.79	64.84 ± 14.40	
Level of education				**p**_**c**_** < 0.001**			p_c_ = 0.900			**p**_**c=**_**0.024**			p_c_ = 0.528
	Lower	42.45 ± 13.15	59.59 ± 15.74	p_i_ = 0.371	61.32 ± 13.57	63.95 ± 14.72	p_i_ = 0.387	51.30 ± 20.23	58.77 ± 16.63	p_i_ = 0.351	63.98 ± 15.96	64.98 ± 13.66	p_i_ = 0.973
	Middle	49.09 ± 15.68	60.60 ± 17.20		63.27 ± 2.27	63.18 ± 15.58		54.00 ± 19.22	59.73 ± 16.15		63.29 ± 14.40	64.53 ± 12.68	
	Higher	48.52 ± 2.68	63.74 ± 17.48		68.84 ± 2.69	66.81 ± 15.28		60.13 ± 26.81	60.48 ± 18.38		68.68 ± 16.33	69.10 ± 15.57	
Job profile				**p**_**c**_** < 0.001**			p_c_ = 0.541			**p**_**c**_** = 0.010**			p_c_ = 0.446
	Manual	41.44 ± 13.93	61.02 ± 16.50	**p**_**i**_** = 0.008**	60.83 ± 13.50	65.56 ± 13.74	**p**_**i**_** = 0.003**	51.98 ± 20.90	58.43 ± 17.57	p_i_ = 0.500	62.91 ± 15.15	65.11 ± 14.18	p_i_ = 0.408
	Non-manual	50.65 ± 15.64	61.06 ± 16.98		66.58 ± 2.50	63.45 ± 16.23		56.73 ± 22.56	60.52 ± 16.21		66.59 ± 15.74	66.50 ± 13.65	
Affected joint				**p**_**c**_** < 0.001**			p_c_ = 0.852			**p**_**c**_** = 0.014**			p_c_ = 0.504
	Knee	50.75 ± 15.64	61.45 ± 17.78	**p**_**i**_** = 0.038**	66.52 ± 15.63	64.37 ± 15.28	p_i_ = 0.075	56.71 ± 22.59	60.50 ± 17.54	p_i_ = 0.568	65.70 ± 17.11	66.38 ± 13.78	p_i_ = 0.859
	Hip	42.80 ± 14.56	60.69 ± 15.81		61.78 ± 14.13	64.42 ± 15.13		52.73 ± 21.21	58.77 ± 16.20		64.27 ± 14.10	65.44 ± 14.00	
Comorbidity				**p**_**c**_** < 0.001**			p_c_ = 0.884			p_c_ = 0.099			p_c_ = 0.721
	Reported	42.00 ± 14.36	55.10 ± 15.73	p_i_ = 0.636	62.20 ± 12.79	60.70 ± 16.37	p_i_ = 0.414	56.67 ± 19.93	57.93 ± 15.44	p_i_ = 0.273	65.93 ± 12.01	62.37 ± 15.19	p_i_ = 0.057
	Not reported	48.01 ± 15.69	63.02 ± 16.62		64.59 ± 15.65	65.63 ± 14.59		53.90 ± 22.54	60.12 ± 17.27		64.60 ± 16.58	67.04 ± 13.25	
BMI(kg/m^2^) category				**p**_**c**_** < 0.001**			p_c_ = 0.467			**p**_**c**_** = 0.014**			p_c_ = 0.960
	18.5–24.9	44.12 ± 15.96	62.18 ± 18.03	p_i_ = 0.479	59.71 ± 11.27	61.18 ± 17.26	p_i_ = 0.262	53.65 ± 21.15	61.06 ± 14.58	p_i_ = 0.816	67.12 ± 9.69	64.06 ± 10.46	p_i_ = 0.497
	25.0–29.9	46.97 ± 16.23	63.22 ± 16.91		64.47 ± 14.39	67.89 ± 14.23		53.44 ± 22.47	59.17 ± 19.11		66.92 ± 16.37	68.72 ± 16.26	
	≥ 30.0	46.87 ± 15.23	59.58 ± 16.35		64.82 ± 16.06	63.34 ± 14.93		55.45 ± 22.01	59.42 ± 16.20		63.31 ± 16.23	64.81 ± 13.13	

p_c_: *p* value for both group’s combined change; p_i_: *p* value of group interaction

#### Disease-specific QOL (WOMAC)

Disease-specific QOL outcome results indicated that overall, patients gained significantly better QOL in all domains of the WOMAC score (Table 4).

**Table 4:** Population results of the disease-specific QOL assessment

WOMAC			
Domain	Preoperative data	Postoperative data	
	mean ± SD	mean ± SD	
Pain	57.03 ± 21.87	28.09 ± 20.87	p < 0.001
Stiffness	59.08 ± 21.28	29.71 ± 22.59	p < 0.001
Physical function	61.26 ± 18.06	34.01 ± 20.61	p < 0.001
Total score	60.19 ± 17.57	32.39 ± 19.84	p < 0.001

In the sub-group analysis (Table 5), participants from the younger age-group (< 65) reported significant decrease in joint stiffness (*p* = 0.005), and overall, a better disease-specific QOL (*p* = 0.05). Patients in the manual group reported significantly greater increase in physical function (*p* = 0.037) and overall score (*p* = 0.024) compared to the non-manual group. Patients with hip OA seemed to gain the most out of their operation as they reported better outcome in the WOMAC pain (*p* = 0.019), stiffness (*p* = 0.010), physical function domains (*p* = 0.011) and total score (*p* = 0.007) compared to knee OA patients. Participants who reported no comorbidities had significant decrease of joint stiffness compared to comorbid patients (*p* = 0.010). Regarding the connection of BMI and QOL, normal weight and overweight patients reported a significant decrease in their pain compared to obese patients (*p* = 0.017).

**Table 5:** Sub-group results of the disease-specific QOL assessment

		WOMAC											
		Pain			Stiffness			Physical function			Total score		
		Preop	Postop	Sig	Preop	Postop	Sig	Preop	Postop	Sig	Preop	Postop	Sig
		mean ± SD	mean ± SD		mean ± SD	mean ± SD		mean ± SD	mean ± SD		mean ± SD	mean ± SD	
Gender				**p**_**c**_** < 0.001**			**p**_**c**_** < 0.001**			**p**_**c**_** < 0.001**			**p**_**c**_** < 0.001**
	Male	55.56 ± 22.97	26.67 ± 20.87	p_i_ = 0.988	61.62 ± 21.64	27.16 ± 20.09	p_i_ = 0.228	60.91 ± 17.49	32.80 ± 22.13	p_i_ = 0.813	59.98 ± 17.56	30.84 ± 21.23	p_i_ = 0.714
	Female	57.70 ± 21.47	28.73 ± 20.97		57.93 ± 21.15	30.85 ± 23.66		61.42 ± 18.41	34.56 ± 20.02		60.28 ± 17.68	33.08 ± 19.28	
Age groups				**p**_**c**_** < 0.001**			**p**_**c**_** < 0.001**			**p**_**c**_** < 0.001**			**p**_**c**_** < 0.001**
	< 65	58.65 ± 19.97	24.39 ± 19.13	p_i_ = 0.244	65.97 ± 20.22	23.39 ± 18.64	**p**_**i**_** = 0.005**	63.45 ± 16.85	28.18 ± 17.08	p_i_ = 0.050	62.66 ± 16.40	26.99 ± 16.70	**p**_**i**_** = 0.050**
	≥ 65	56.45 ± 22.61	29.44 ± 21.42		56.65 ± 21.23	31.93 ± 23.52		60.50 ± 18.49	36.05 ± 21.42		59.29 ± 17.97	34.34 ± 20.54	
Level of education				**p**_**c**_** < 0.001**			**p**_**c**_** < 0.001**			**p**_**c**_** < 0.001**			**p**_**c**_** < 0.001**
	Lower	63.81 ± 24.16	31.19 ± 22.05	p_i_ = 0.604	63.98 ± 24.63	32.61 ± 25.71	p_i_ = 0.866	68.60 ± 17.35	38.26 ± 21.16	p_i_ = 0.514	67.40 ± 17.96	35.75 ± 21.02	p_i_ = 0.445
	Middle	52.82 ± 19.16	26.14 ± 20.17		55.67 ± 18.11	27.44 ± 20.77		59.24 ± 14.47	32.16 ± 20.05		57.37 ± 14.22	30.58 ± 18.90	
	Higher	53.73 ± 20.56	26.60 ± 20.37		57.00 ± 19.68	28.83 ± 20.50		53.78 ± 20.29	30.66 ± 20.26		54.31 ± 18.66	30.39 ± 19.54	
Job profile				**p**_**c**_** < 0.001**			**p**_**c**_** < 0.001**			**p**_**c**_** < 0.001**			**p**_**c**_** < 0.001**
	Manual	62.12 ± 23.82	28.35 ± 20.41	p_i_ = 0.114	63.80 ± 23.63	29.26 ± 23.16	p_i_ = 0.096	67.81 ± 15.71	34.99 ± 19.79	**p**_**i**_** = 0.037**	66.42 ± 16.70	32.56 ± 19.37	**p**_**i**_** = 0.024**
	Non-manual	52.91 ± 19.37	27.88 ± 21.40		55.15 ± 18.39	30.08 ± 22.28		55.90 ± 18.18	33.22 ± 21.37		55.20 ± 16.74	32.25 ± 20.35	
Affected joint				**p**_**c**_** < 0.001**			**p**_**c**_** < 0.001**			**p**_**c**_** < 0.001**			**p**_**c**_** < 0.001**
	Knee	56.48 ± 23.07	34.41 ± 23.54	**p**_**i**_** = 0.019**	58.04 ± 20.51	36.95 ± 25.11	**p**_**i**_** = 0.010**	59.84 ± 17.84	39.14 ± 23.44	**p**_**i**_** = 0.011**	58.85 ± 17.57	37.96 ± 22.78	**p**_**i**_** = 0.007**
	Hip	57.52 ± 20.95	22.58 ± 16.55		60.00 ± 22.07	23.25 ± 17.94		62.50 ± 18.29	29.53 ± 16.71		61.37 ± 17.62	27.45 ± 15.36	
Comorbidity				**p**_**c**_** < 0.001**			**p**_**c**_** < 0.001**			**p**_**c**_** < 0.001**			**p**_**c**_** < 0.001**
	Reported	59.93 ± 20.47	36.97 ± 25.09	p_i_ = 0.209	54.14 ± 21.38	37.59 ± 27.21	**p**_**i**_** = 0.010**	61.98 ± 18.73	39.63 ± 23.21	p_i_ = 0.245	61.38 ± 17.85	39.87 ± 22.49	p_i_ = 0.136
	Not reported	56.07 ± 22.34	25.13 ± 18.50		60.67 ± 21.12	27.17 ± 20.42		61.02 ± 17.93	32.14 ± 19.45		59.79 ± 17.56	29.92 ± 18.36	
BMI (kg/m^2^) category				**p**_**c**_** < 0.001**			**p**_**c**_** < 0.001**			**p**_**c**_** < 0.001**			**p**_**c**_** < 0.001**
	18.5–24.9	66.11 ± 19.35	22.82 ± 17.90	**p**_**i**_** = 0.017**	55.00 ± 21.29	25.88 ± 20.93	p_i_ = 0.088	58.27 ± 22.96	28.27 ± 15.23	p_i_ = 0.127	59.63 ± 20.13	26.94 ± 15.46	p_i_ = 0.074
	25.0–29.9	55.06 ± 22.94	20.75 ± 14.96		61.43 ± 19.95	22.71 ± 17.63		61.68 ± 16.20	27.84 ± 17.83		60.29 ± 16.77	25.44 ± 15.42	
	≥ 30.0	55.67 ± 21.69	32.93 ± 22.79		58.88 ± 22.08	34.33 ± 24.38		61.79 ± 17.84	38.79 ± 22.11		60.27 ± 17.54	37.20 ± 21.52	

*p*_c_: *p* value for both group’s combined change; *p*_*i*_: *p* value of group interaction

Total WOMAC score indicated significantly better disease-specific QOL post-surgery in all subgroups, regardless of the patients’ gender, level of education, BMI category or the presence of other diseases (p < 0.001).

MacKay et al. carried out a literary review of 13 articles assessing the minimal clinically important difference (MCID) of WOMAC in patients who underwent total hip or knee replacement, and reported a MCID between 8.3–41 and 9.7–34 for pain and function domain respectively. For knee replacement they reported a MCID between 13.3–36 and 1.8–33 for pain and function domain respectively [33].

If we accept the value of MCID as 8.3 for pain and 9.7 for function domain, 83.87% and 81.25% of our hip OA patients exceeded these values. If we also accept the lowest reported MCID values for knee OA patients, 66.67% and 76.79% of our knee patients exceeded these values respectively.

## Discussion

In this study, we aimed to investigate the general and disease-specific QOL of osteoarthritic patients pre- and post-surgery, and whether surgery would result in significant improvement. Disease-specific measures are considered to be more accurate for assessing immediate effects, whereas generic measures might reveal effects of the surgery in the long run as observed by Neuprez et al. [34].

As reported by other studies, domains related to physical health status show relatively lower scores as compared with psychological components in patients before surgery [35]; this result is consistent with our results.

The results indicated statistically significant improvement in all domains concerning physical functions in the study population as well as in the domain of social relationships, the latter indicating the impact of the difficulty of getting around in one’s environment.

In this study, we investigated the possible role of independent variables in the disease-specific disability functional assessment performed using the WOMAC questionnaire. According to our results, patients with hip OA seemed to gain the most out of their operation, as they reported better outcome in the WOMAC pain, stiffness, physical function domains and total score compared to knee OA patients. A similarly significant improvement was measured among participants of the working age group (< 65 years) compared to the older patients and with manual jobs compared to non-manual workers as shown in Table 5. As the latter group is heavily exposed to the degeneration of cartilage because of their profession, it is a significant achievement that they can benefit from the surgery to this extent. The success of the surgery among the working age population indicates that many of them may be able to return to their job actively, thus decreasing the economic burden of OA.

Our results regarding the effect of the surgery on QOL outcomes are consistent with other studies. Papakostidou et al. found that after TKA, all groups of patients showed a statistically significant improvement in WOMAC domains between the pre- and the 12-month post-operative assessments, and there have been no significant differences in WOMAC domains in age, BMI, education and gender [36].

On the contrary, other studies have shown opposite results regarding the effect of gender and BMI. In a pooled analysis of 1783 knee and 2400 hip OA patients, Hofstede et al. have reported that being female or having higher BMI are associated with lower postoperative HRQoL and functioning and more pain [37]. Alkan et al. [38] have also found that WOMAC pain scores are higher in female patients; however, we found no difference between the two genders either pre- nor post-surgery. It has been reported that over 50% of the patients who required total knee replacement for end-stage OA are obese [39], a ratio that we experienced as well. However, we saw no difference in improvement by BMI category either in the generic or in the disease specific QOL.

Even though the success of joint replacement surgery is indisputable, additional therapies have been shown to boost its efficacy. The results of a study by Desmeules et al. suggest that the prehabilitation programme not only can alter the physical decline caused by OA, but it can help participants to improve their level of function before surgery as well, which is an important achievement in view of the fact that preoperative physical function is a major determinant of postoperative physical function [40, 41].

Although several studies investigated QOL among patients with OA, the cornerstone of those were a disease-specific QOL instrument. To assess the general QOL, many studies chose the 36-Item Short Form Survey which do measure aspects that are linked to health and functional performance. However, WHOQOL-BREF has been shown to be a better fit for assessing global QOL and thus a better choice to gain a more comprehensive picture when investigating QOL, which we aimed to contribute the literature.

## Conclusion

OA was proved to cause severe pain and disability regardless of the patients’ socioeconomic and anthropometric characteristics. The results of the physical domain of the general QOL questionnaire were consistent with the results of the disease-specific measures, and thus proved to be sensitive to the physical symptoms caused by the disease. Total hip replacement is regarded as one of the most successful surgeries in medicine today, which is supported by the results of our disease-specific QOL assessment as well as by the physical health domain of the general QOL outcomes. This success was most pronounced in case of the active population and patients doing physical labour. The fact that patients reported a significant improvement in the social relationships domain may indicate that OA itself as well as its management has a profound effect on patients’ life beyond the reduction of their pain.

### Limitations

As data collection was carried out by self-administered questionnaires, inaccuracies in patients’ memories have the potential to distort our data. The limitations of our study are consistent with the nature of observational studies and the bias on patient selection, for which we tried to correct by selecting a large number of participants from two different counties of the country and by enrolling them consecutively. Although this study was carried out in two different health centres, both of them were from the South-Eastern region of Hungary, and so findings may not be generalizable to the overall Hungarian population. Also, as the study population only consisted of patients with severe OA, we cannot extrapolate our results to patients with mild or moderate OA. Postoperative data collection was carried out after the local outbreak of the COVID-19 pandemic, which might alter the outcome data, especially that of the psychological domain.

## Data Availability

The datasets analysed during the current study are available from the corresponding author on reasonable request.

## Abbreviations

BMI: Body mass index
HRQOL: Health-related Quality of life
MCID: Minimal clinically important difference
OA: Osteoarthritis
OARSI: Osteoarthritis Research Society International
OMERACT: Outcome Measures in Rheumatoid Arthritis Clinical Trials group
QOL: Quality of life
SD: Standard deviation
SF-36: 36-Item Short Form Survey
SPSS: Statistical Package for the Social Sciences
WHOQOL: World Health Organization Quality of life generic measurement tool
WHOQOL-BREF: 26-Item World Health Organization Quality of Life questionnaire
WOMAC: Western Ontario and McMaster Universities Osteoarthritis Index
